# Bibliometric Analysis of Artificial Intelligence in Pediatric Radiology and Medical Imaging: A Focus on Deep Learning Applications

**DOI:** 10.3390/bioengineering13040461

**Published:** 2026-04-14

**Authors:** Ahmad Tijjani Garba, Aminu Bashir Suleiman, Wenze Du, Ahmed Ibrahim Mahmud, Harisu Abdullahi Shehu, Huseyin Kusetogullari, Md. Haidar Sharif

**Affiliations:** 1 Department of Information Technology, Bayero University, Kano, P.M.B. 3011, Kano 700241, Nigeria; atgarba.it@buk.edu.ng; 2 Department of Cyber Security, Federal University Dutsin-Ma, P.M.B. 5001, Dutsin-Ma 821101, Katsina, Nigeria; 3 College of Intelligence and Computing, Tianjin University, Tianjin 300350, China; 4 School of Mathematics, Tianjin University, Tianjin 300350, China; duwenze_096@tju.edu.cn; 5 Department of Software Engineering, Federal University Dutsin-Ma, P.M.B. 5001, Dutsin-Ma 821101, Katsina, Nigeria; amidot2005@gmail.com; 6 School of Engineering and Computer Science, Victoria University of Wellington, Wellington 6012, New Zealand; harisushehu@ecs.vuw.ac.nz; 7 Department of Computer Science, Blekinge Institute of Technology, 37141 Karlskrona, Sweden; huseyin.kusetogullari@bth.se; 8 Department of Computer Science, Capitol Technology University, Laurel, MD 20708, USA; hsharif@captechu.edu

**Keywords:** pediatric radiology, artificial intelligence, deep learning, thematic mapping, bibliometric analysis

## Abstract

This study presents the first dedicated bibliometric analysis of artificial intelligence (AI) and deep learning applications in pediatric radiology and medical imaging, mapping the intellectual structure of a rapidly evolving field. A total of 2688 articles and conference proceedings published between 2005 and 2025 were retrieved from the Web of Science Core Collection and analyzed using Bibliometrix R and VOSviewer. The findings reveal exponential growth in publications, from 7 papers in 2005 to 559 in 2025, with journal articles dominating the corpus (85.9%). The most-cited contributions, led by Kermany et al. (2018) with 2886 citations, are predominantly technical feasibility studies rather than clinical outcome trials, indicating a field that has advanced methodologically but remains in early stages of clinical translation. Thematic mapping identifies convolutional neural networks, pneumonia, and transfer learning as Motor Themes representing methodological maturity in chest imaging, while neuroimaging and image segmentation clusters occupy Niche Themes, reflecting insular development with limited cross-field connectivity. Geographic analysis reveals concentrated co-authorship along US–China and US–Europe corridors, with African, Latin American, and Southeast Asian institutions largely absent from knowledge production networks. Eight of the ten most productive affiliations are North American, highlighting structural inequities that risk producing AI tools optimized for high-resource settings rather than the global pediatric population. This analysis provides an empirical foundation for reorienting the field toward clinical validation, geographic inclusion, and methodological integration across isolated research communities.

## 1. Introduction

Artificial intelligence and deep learning applications in the radiology field have developed significantly over the last forty years, starting with crude rule-based solutions and advancing to complex data-driven neural networks [1,2,3]. Early experiments on computer-based detection demonstrated the possibilities of automating the interpretation of images, especially in mammographic screening, but early systems were limited both by manual feature extraction and standard classifiers, and they continued to achieve vastly high false positive rates [4,5,6,7]. One of the most significant changes happened after 2012, when convolutional neural networks demonstrated significantly better results in large-scale visual tasks due to the presence of large amounts of labeled data and the development of graphics processing units [8]. This was further supported by the post-collection of large repositories of medical images and carefully collected benchmark datasets, which led to the clinical translation of these methods [9]. The current research hence falls under this historical flow, with emphasis on the fact that methodological advancements have introduced new frontiers in diagnostic imaging.

Pediatric radiology is a critical domain where timely and accurate imaging can decisively influence clinical outcomes in children. Unlike adults, children have unique anatomy, physiology, and disease patterns, and they are more vulnerable to the risks of ionizing radiation [10,11]. AI and deep learning technologies hold particular promise in this setting by augmenting human expertise in image interpretation and decision-making. For example, AI-driven image analysis has demonstrated expert-level pattern recognition, improving the sensitivity of detecting subtle findings and reducing diagnostic errors in pediatric scans [12,13]. Deep learning constitutes a specific subset of the broader artificial intelligence paradigm, representing a family of multi-layered neural network architectures that learn hierarchical feature representations directly from raw data [14]. Furthermore, advanced deep learning image reconstruction allows high-quality imaging at significantly lower radiation doses. Studies show dose reductions of 36–70% in pediatric CT without loss of diagnostic information [15]. AI can also accelerate image acquisition and processing (e.g., faster MRI sequences and automated measurements), which improves workflow efficiency and helps avoid sedation or delays [16,17]. By enhancing diagnostic accuracy, minimizing radiation exposure, expediting image interpretation, and providing clinical decision support, AI has the potential to markedly improve the safety and quality of pediatric imaging care [18].

Recent years have witnessed an explosive growth of research on artificial intelligence in pediatric medical imaging. Following the broader radiology trends [19,20], pediatric imaging AI publications have surged across a variety of modalities and applications. Deep learning approaches are being applied to all major pediatric imaging modalities, including radiography, ultrasound, CT, MRI, and nuclear medicine for tasks ranging from anomaly detection and disease classification to segmentation and image enhancement [21]. These efforts span numerous pediatric conditions and subspecialties: for instance, dedicated algorithms now assist in everything from diagnosing pediatric pneumonia on chest X-rays to detecting pediatric brain tumors and musculoskeletal injuries [22,23]. The diversity of algorithms and use cases is immense, reflecting how AI methods are rapidly permeating pediatric radiology. However, this rapid expansion has also led to fragmentation. Research outputs are often siloed by imaging modality or clinical focus, with little crossover between subfields [11,24]. In practice, the majority of commercial and academic AI solutions primarily focus on adult imaging, with only a small number specifically catering to pediatric needs [25]. This fragmented landscape makes it challenging to obtain a cohesive view of all AI developments in pediatric radiology, indicating the importance of an integrative analysis of the field.

Several narrative and systematic reviews of AI applications in pediatric imaging have appeared, but they tend to be limited in scope. Many focus on a single modality or subspecialty; for example, dedicated overviews exist for pediatric neuroradiology [11,26], pediatric musculoskeletal imaging (e.g., hip disorders) [27], and primers on AI for pediatric radiologists [11]. While such reviews provide valuable clinical insights, they do not offer a longitudinal or structural perspective on the field as a whole. Traditional reviews generally present a static snapshot of current applications, without analyzing how research trends have evolved or how different research topics interconnect. Crucially, they lack science-mapping analyses: conventional reviews do not examine collaboration networks, citation patterns, or other bibliometric indicators that reveal the domain’s intellectual structure. Important questions, therefore, remain unaddressed. For instance, existing reviews do not identify which institutions or countries are driving the most research, how clusters of topics (e.g., “deep learning in fetal imaging” or “AI for pediatric tumors”) relate to each other, or how the focus of pediatric imaging AI has shifted over the years. In short, the literature to date, though rich in clinical detail, has not provided a comprehensive, data-driven map of this rapidly evolving field [20,28]. This gap motivates the need for a different analytical approach.

Bibliometric analysis complements these limitations. Bibliographic analysis can reveal hidden patterns and linkages in the literature that standard assessments cannot [29]. Co-citation and co-authorship network analysis can reveal the field’s intellectual structure, significant research subjects, how they cluster, and which papers or authors serve as “nodes” between subfields [30]. Bibliographic analysis of keyword co-occurrence and citation bursts can identify research hotspots and emerging trends, such as “radiation dose reduction,” “fetal MRI,” and “COVID-19 applications,” and reveal when specific topics gained popularity. This method also ranks prolific authors, leading institutions, and high-impact publications in pediatric imaging AI to trace seminal work and analyze worldwide collaboration trends [31]. Bibliometric studies use a macroscopic, longitudinal perspective to show how radiology-computer science collaborations have changed, and how research has shifted from bone age assessment to federated learning [30]. In conclusion, bibliometric analysis quantitatively maps the knowledge ecosystem rather than qualitatively summarizing clinical findings to provide a “science map” of the subject, giving strategic insights into its evolution.

A review of the existing literature confirms that while bibliometric analyses of artificial intelligence (AI) in general medical imaging have emerged, to the best of our knowledge, no dedicated bibliometric study has yet been conducted on the specific and exact application of AI and deep learning (DL) in pediatric radiology and medical imaging. This study, therefore, addresses a clear and significant gap by providing the first focused, microscopic bibliometric examination of this specialized and critical domain. Through detailed visual mapping and network analysis, this work will establish a foundational overview of the field, delineating key research achievements to help scholars, clinicians, and policymakers identify influential authors, core journals, leading countries, affiliations, seminal references, and evolving thematic trends. A co-authorship network analysis will offer unprecedented insights into the intellectual collaboration patterns driving innovation in pediatric AI imaging. Specifically, the paper will: (i) analyze publication and citation growth from a defined period from 2005–2025; (ii) map co-authorship networks across countries, authors, and institutions; and (iii) examine the global co-occurrence network of author keywords to uncover research fronts. It is anticipated that these findings will not only fill a critical knowledge void but also stimulate targeted future research, ultimately benefiting pediatric patients and healthcare providers by mapping the field’s structure and opportunities. This study is designed to serve as an essential reference guide for both new and established researchers interested in the potential of AI/DL for pediatric image interpretation, highlighting active contributors and significant topics in an otherwise unmapped landscape. The analysis will specifically trace the growing integration of deep learning techniques across the broader AI landscape, recognizing their transformative impact on pediatric diagnostic imaging. Based on this inaugural analysis, we project that AI and deep learning will continue to evolve as pivotal, yet underexplored, tools in pediatric radiology, warranting and guiding focused scholarly attention in the years ahead.

## 2. Materials and Methods

The dataset for this bibliometric study was obtained from the Web of Science Core Collection (WoSCC) and downloaded on 5 January 2026. Records were retrieved using a Topic Search (TS) query built around three concept blocks (AI methods, pediatric population, and medical imaging), combined with AND operators as shown in Table 1.

All records were exported in both BibTeX formats to preserve complete bibliographic information and cited reference data.

To be included in studies, the research needed to (i) be indexed in the Web of Science Core Collection (WoSCC)/Science Citation Index Expanded (SCIE) within the specified time range and (ii) report an application of artificial intelligence/machine learning/deep learning to pediatric populations in radiology or medical imaging, with relevant modalities (such as computed tomography, magnetic resonance imaging, ultrasound, or X-ray) and/or imaging tasks (such as segmentation, detection, classification, or radiomics). To meet the exclusion criteria, the studies were eliminated provided that they (i) were not written in English; (ii) were not published after 2005 or before 2025; (iii) were not excluded due to their publication in other document types, such as review, editorial material, letter, note, meeting abstract, or book chapter; or (iv) failed to meet all three conceptual criteria, which included the use of AI methods, a pediatric focus, and an imaging/radiology setting, based on the title/abstract. This also included literature that was restricted to adult imaging, non-imaging clinical AI (e.g., purely electronic health record analyses), and papers about the development of methods which did not include a clear application of pediatric medical imaging.

**Figure 1:**
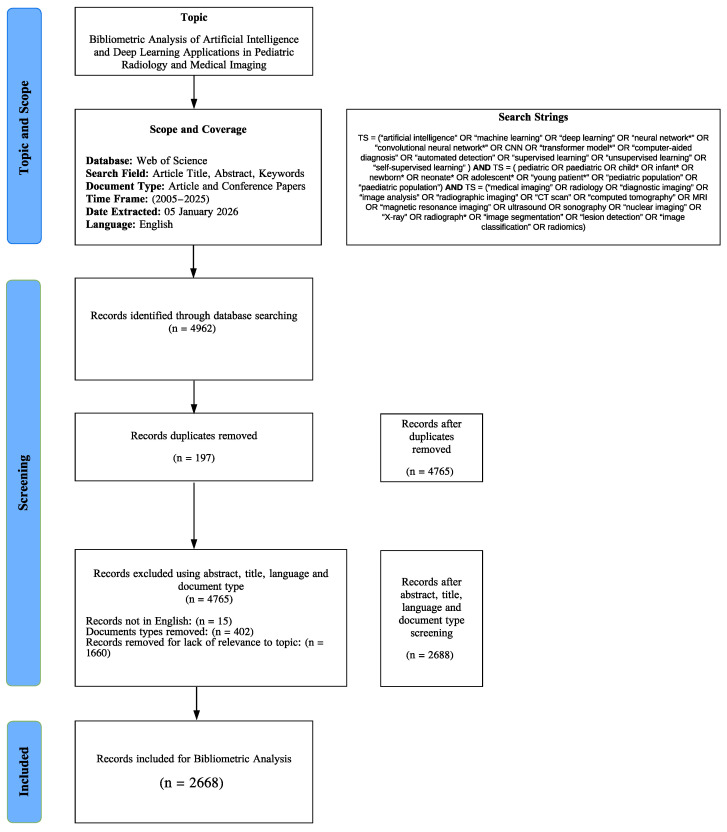
Research process flow diagram.

4962 records were preliminarily identified. Duplicate elimination was done by a hierarchical key of DOI, then the WoS accession number and finally a normalized title, thereby removing 197 duplicates and leaving 4765 distinct records. Then, the eligibility filters based on the scope of the study were implemented: the English language filter removed 15 records, which left 4750; the document-type filter, which is Articles and Conference Proceedings, eliminated 402 records, and the remaining records were 4348. Relevance in Titles and abstracts was filtered with the help of pre-existing blocks of keywords, which included AI/ML/DL terms, pediatric terms, and medical imaging/radiology terms. This selection stage excluded 1660 records that were found to be irrelevant, and 2688 studies were obtained to be included in the final bibliometric analysis, as shown in Figure 1.

Core bibliographic fields of country/region, authors, institutions, journal title, reference list and keywords were taken out of each record that passed inclusion criteria and were to be further analysed. VOSviewer 1.6.20 [32] was used to perform bibliometric mapping and visualization in order to identify patterns of cited authorship. The analysis and keyword-based visualizations were done in Bibliometrix-RStudio [33] and helped to identify the research hotspots, the development of the theme, and the emergence of the trends in the field of AI and deep learning application in pediatric radiology and medical imaging.

### Thematic Mapping Methodology

The thematic map employed in this study is a bibliometric visualization tool originally introduced by [34] and implemented within the Bibliometrix R package. It positions keyword clusters on a two-dimensional plane defined by two quantitative metrics: centrality and density. Centrality, represented on the x-axis, measures the degree of interaction between a given cluster and all other clusters in the keyword co-occurrence network, calculated as the sum of co-occurrence link weights between keywords belonging to a given cluster and keywords belonging to all other clusters in the network. A high centrality score indicates that a theme is strongly connected to the broader research landscape and plays an important structural role in the overall knowledge base of the field. Density, represented on the y-axis, measures the internal cohesion of a cluster, specifically the strength of co-occurrence links among keywords within the same cluster, with a high density score indicating that the keywords within a cluster frequently co-occur with one another and therefore form a well-developed, internally coherent research theme. The intersection of these two metrics produces four interpretive quadrants: Motor Themes, located in the top-right, represent themes that are both highly central and highly dense, constituting the well-developed intellectual backbone of the field; Niche Themes, in the top-left, are internally developed but peripheral, representing specialized subfields with limited cross-field connectivity; Basic Themes, in the bottom-right, are broadly relevant but loosely developed, functioning as foundational umbrella concepts rather than distinct research directions; and Emerging or Declining Themes, in the bottom-left, represent marginal or transitional topics that are either newly entering the literature or gradually losing scholarly momentum.

The thematic map employed in this study is a rigorous bibliometric visualization tool originally conceptualized by [34] and implemented via the ‘thematicMap()’ function in the Bibliometrix v4.x package within the R Studio programming V2026.01.0 environment, an established gold standard for bibliometric science mapping. This methodology positions keyword clusters on a two-dimensional plane defined by two core quantitative metrics with precise mathematical and operational definitions: centrality (x-axis), the total sum of co-occurrence link weights between a cluster’s keywords and all other clusters in the network, which quantifies inter-theme interaction and structural relevance to the broader research landscape, and density (y-axis), the weighted mean of co-occurrence link strengths among a cluster’s internal keywords, normalized to a 0–100 scale for intuitive comparative analysis, which quantifies a cluster’s internal cohesion and developmental maturity. Prior to analysis, comprehensive author keyword (field tag: DE) normalization was conducted to eliminate semantic redundancy, including systematic synonym consolidation and unification of variant forms (e.g., ‘MRI’ → ‘magnetic resonance imaging’, ‘CNN’ → ‘convolutional neural network’, ‘deep-learning’ → ‘deep learning’, ‘paediatric’ → ‘pediatric’). A minimum keyword co-occurrence frequency threshold of 5 was enforced, retaining only terms appearing in at least 5 documents to focus analysis on meaningful, recurring thematic connections rather than isolated, low-relevance terms.

## 3. Results and Analysis

### 3.1. Analysis Document Types

**Figure 2:**
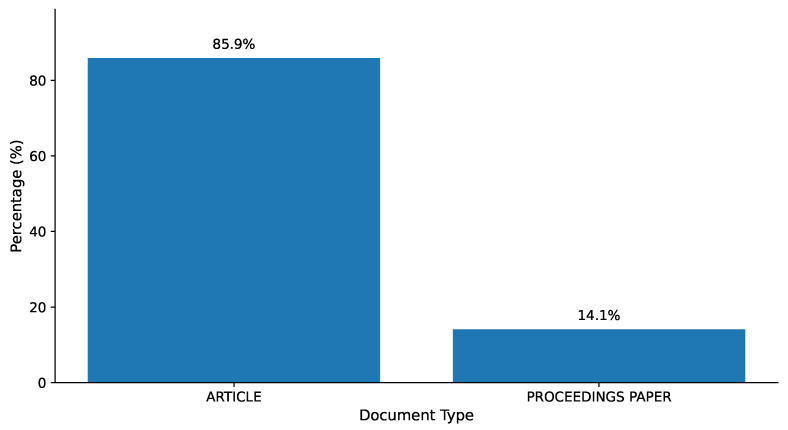
Document Type Distribution.

The document type analysis of the bibliometric dataset, as illustrated in Figure 2, reveals that the 2688 publications are 85.9 percent peer-reviewed papers and 14.1 percent proceedings articles. This shows that the discipline favors high-quality, peer-reviewed research in reputable journals. The comparatively low proportion of conference proceedings indicates that new interdisciplinary research might be underrepresented in such conferences, creating a disparity between the accurate reflection of the rate of innovation in the field.

### 3.2. Growth of Scientific Production

**Figure 3:**
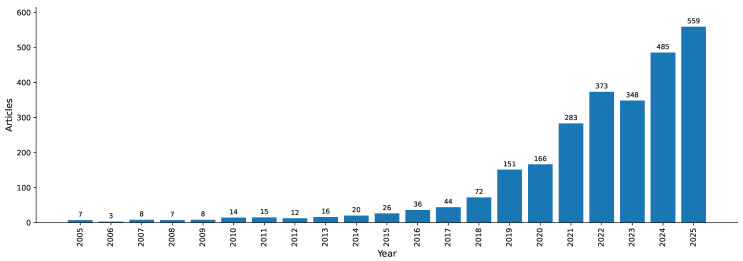
Annual Scientific Production.

The trend in the number of scientific publications on AI applications in pediatric radiology and medical imaging shows significant exponential growth between 2005 and 2025, as shown in Figure 3. The number of published papers was only 7 in 2005; it decreased to 3 in 2006, and annual production remained below 20 until 2016. Since then, the rate has increased by a factor of four: 151 in 2020, 283 in 2022, 373 in 2023, 348 in 2024, and finally 559 in 2025. The initial sluggishness was due to the fact that there was less information and computing capabilities to structure pediatric populations. The increase after 2018 is associated with increased interdisciplinary cooperation, clinical validation and the fourfold increase in the volume of publications in general medical AI.

### 3.3. Most Relevant Sources

The bibliometric analysis in Table 2 reveals that the first source in terms of the number of publications is Scientific Reports (67), then Pediatric Radiology (61), Neuroradiology (54), PLOS ONE (50), IEEE Access (47), Diagnostics (39), Biomedical Signal Processing and Control (31), Frontiers in Pediatrics (29), Human Brain Mapping (30), and Frontiers in Neuroscience (30). This indicates a wide range of interdisciplinary and specialized journals that can contribute to the development of AI in Pediatric imaging. Distribution also shows that open-access sources, including Scientific Reports and PLOS ONE, are the most popular in spreading empirical research on deep learning applications. Pediatric Radiology and Neuroradiology are examples of specialized outlets that emphasize contributions to clinical integration. Interdisciplinary collaborations are indicated in engineering-oriented journals like IEEE Access and Biomedical Signal Processing and Control. These Journals focus on algorithmic advancements of signal processing and anomaly detection.

### 3.4. Most Relevant Authors

Table 3 presents the ten most productive author name signatures in the corpus, revealing a highly concentrated publication landscape. Zhang Y leads with 68 documents, followed by Wang Y (60) and Wang L (50), establishing a pronounced productivity gap between the top three contributors and the remainder of the list. A second tier of consistently publishing authors, Li J (47), Li H (45), and Liu Y (45), reflects sustained engagement with the field rather than isolated contributions, while Wang J (43), Zhang J (42), Li Y (38), and Chen Y (37) complete the top ten with substantial individual volumes. This distribution is consistent with Bradford’s law of scattering, whereby a small nucleus of highly productive authors account for a disproportionate share of the total output in a given field [34]. The dominance of East Asian name signatures, particularly Chinese surnames, is consistent with the country-level finding that China accounts for the largest total citation count in the corpus (8804 TC), reflecting the broader national investment in healthcare AI infrastructure that has made China the world’s leading producer of AI-related medical research since approximately 2017 [20]. The presence of the same surnames across multiple high-frequency entries (multiple “Wang,” “Zhang,” and “Li” signatures in the top ten) suggests that large, recurring research consortia rather than purely individual investigators are responsible for a significant share of the output. This pattern implies thematic and methodological continuity: recurring author groups reinforce the dominance of specific CNN-based architectures and imaging modalities (MRI, CT) across the literature, creating a self-reinforcing citation ecosystem around established methodological pipelines.

It is important to note that author name analysis at the name signature level, as produced by the WoSCC surname-initial indexing convention, has inherent disambiguation limitations. The format “Zhang Y” cannot distinguish between Zhang Yan, Zhang Yang, Zhang Yue, or any other researcher sharing that initial combination. This is a well-documented challenge in bibliometric studies and is particularly acute in research communities with high concentrations of shared family names [35]. The figures in Table 3 should therefore be interpreted as productivity estimates at the name signature level rather than as verified individual author outputs. It is also noted that the ninth-ranked entry (“Wang,” with no given name initial) reflects an incomplete name field in the raw WoSCC export and represents an aggregation artifact rather than a single identifiable researcher.

### 3.5. Most Cited Documents

In Table 4, the most-cited documents in the dataset exhibit a strongly skewed citation distribution, with a small number of papers accounting for a substantial share of total citations. As Ref. [36] is the clear outlier with 2886 citations, far ahead of the rest. The next most cited works are [37] (616 citations) and [38](572 citations), followed by a mid-high tier led by [39] (435), [40] (417 citations), and [41] (404). This pattern suggests that a few foundational contributions dominate the dataset’s intellectual base. Beyond the top few, citations remain substantial but taper gradually across the remaining highly cited papers. The next group includes [42] (374), and two papers tied at 330 citations [43,44], followed by [45] (313) and [46] (304). The rest of the list remains consistently influential: [47] (298), [48] (278), [49] (261), and [50] (258), indicating a broad set of frequently referenced works that collectively shape the field’s most cited knowledge base within this corpus.

### 3.6. Country Collaboration

**Figure 4:**
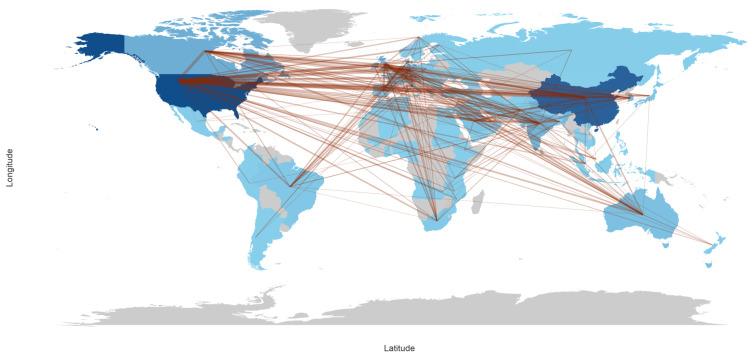
Country Collaboration Map.

The country collaboration map in Figure 4 shows a strongly international co-authorship network, with the densest collaboration links concentrated across the Northern Hemisphere. Two clear hubs stand out: the United States and China, each connected by many lines to multiple regions, indicating broad cross-border partnering rather than primarily domestic collaboration. A third visible hub is Australia, which shows repeated long-distance links, especially into the Eurasian region. The tightest “knot” of intersecting connections appears over Europe, reflecting dense collaboration activity both within Europe and between Europe and the major hubs in North America and East Asia. Overall, the map suggests that collaborations are not evenly distributed: they are most concentrated among high-output, globally connected countries and along the European corridor, with fewer, more scattered links involving Africa and Latin America (where links exist but are visibly less dense). In practical terms, this pattern is typical of a field where large multi-institutional projects, shared datasets, and international clinical/technical partnerships tend to cluster around established research centers. At the same time, other regions participate through fewer cross-border ties.

### 3.7. Most Productive Countries

Table 5 summarizes the national distribution of publications and citations across the ten most productive countries, revealing a field whose knowledge production is concentrated in a small number of high-income economies. China dominates in absolute citation volume with 8804 total citations, reflecting the country’s emergence as the world’s largest producer of AI-related medical research, driven by substantial national investment through programs such as the New Generation Artificial Intelligence Development Plan and access to large-scale clinical imaging infrastructure. However, China’s average citation rate of 13.10 per article, the lowest among the top five, indicates that while Chinese output is volumetrically prolific, a significant portion is concentrated in technical algorithm development studies whose international uptake is more limited than their sheer volume suggests. This pattern is consistent with findings from broader medical AI bibliometric analyses showing that high-volume national outputs do not always translate into proportionally high per-article impact [20,30]. The United States presents a striking inverse relationship: despite ranking only fifth in total citations (1782 TC), it achieves the highest average citation rate in the entire dataset at 26.10 citations per article. This indicates that U.S.-affiliated publications are selectively high-impact, consistent with their concentration on landmark methodological contributions, benchmark dataset releases, foundational architecture papers, and large multicenter clinical validation studies that attract wide international citation. Papers such as [36,46], both originating from U.S. institutions, exemplify this pattern, with citation counts of 2886 and 304, respectively, that disproportionately shape the field’s intellectual base. The United Kingdom and Germany share an identical average citation rate of 22.30, reflecting the strength of European academic-clinical collaborations, particularly in MRI-based neuroimaging and dose optimization research, where both countries have well-established multicenter imaging consortia. Canada (15.00 avg.) maintains consistent output through a combination of strong pediatric hospital networks, including the Hospital for Sick Children in Toronto, and productive cross-Atlantic collaborations. Australia (21.00 avg.) achieves a relatively high per-article impact despite modest total output, suggesting a small but precisely targeted research portfolio. France (18.10) and Spain (18.00) occupy the lower tier of Western European contributors, with both countries sustaining moderate but stable output. Korea (11.30 avg.) and India (9.30 avg.) represent the two largest emerging contributors from Asia. Both countries show lower average citation rates attributable to relatively recent entry into deep learning-based pediatric imaging research and documented constraints in access to large-scale, annotated pediatric imaging datasets of sufficient size and diversity for high-impact studies. The near-total absence of African, Middle Eastern, and Latin American countries from the top-ten list represents a structural gap of significant concern. These regions collectively bear a disproportionate burden of pediatric disease, yet contribute negligibly to the AI literature designed to address it, creating a structural misalignment between where the knowledge is produced and where it is most urgently needed.

### 3.8. Top 10 Most Relevant Affiliations

The institutional distribution presented in Table 6 reveals a marked concentration of output within a small number of North American academic medical centers, with eight of the top ten affiliations located in the United States and Canada. Harvard Medical School leads with 186 articles, followed by the University of Toronto (165), Stanford University (164), and the University of Pennsylvania (156). This clustering reflects the co-location of large pediatric patient populations, world-class computational infrastructure, and deep clinical-research integration at these institutions, which translates directly into high-volume, high-quality publication output. The Children’s Hospital of Philadelphia (122 articles) is particularly notable as the only dedicated pediatric hospital in the top ten, underscoring the role that specialist pediatric institutions play as clinical validation hubs for AI tools developed at adjacent research universities. Its relatively lower article count compared to research-intensive universities reflects a publication strategy oriented toward prospective clinical validation and translational impact rather than toward algorithm development volume. The University of Calgary (115) and Washington University (103) represent major North American neuroimaging centers whose output is concentrated in MRI-based developmental pediatrics, consistent with the thematic map finding that MRI and neuroimaging constitute a well-developed niche cluster in the field. The University of North Carolina (99) is internationally recognized for its longitudinal neuroimaging studies in infant and toddler populations, and its presence in the top ten reflects the sustained influence of its Baby Connectome and similar developmental imaging initiatives on the broader pediatric AI literature. The presence of two Chinese institutions, Shanghai Jiao Tong University (106) and Zhejiang University (106), tied at sixth position, reflects China’s national investment in healthcare AI infrastructure, with both universities contributing predominantly to algorithm development and image segmentation studies, consistent with the country-level finding that Chinese output is technically prolific but concentrated in computational rather than clinical translation literature. A notable absence from the top ten is any European institution, despite the United Kingdom and Germany ranking second and third by total national citations. This likely reflects citation dilution across a larger number of contributing European institutions, none of which individually reaches the output thresholds of the North American leaders. This institutional fragmentation in Europe contrasts with the more concentrated output profile of North American centers and suggests that, despite its high per-article impact, European research may benefit from greater formal consortium-level coordination to consolidate its influence in the global field.

### 3.9. Author Keyword Co-Occurrence

**Figure 5:**
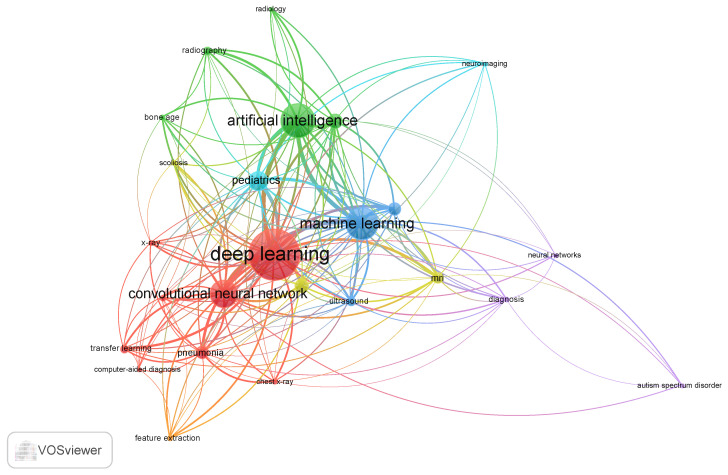
AuthorKeyword Co-occurrence.

Figure 5 describes the author keyword co-occurrence network across four interconnected observations, which was generated using VOSviewer with a label scaling size = 1.8, zoom parameter of 120%, and minimum label display threshold of co-occurrence frequency ≥ 3. The network was constructed using a minimum co-occurrence threshold of 5, yielding 22 keywords organized into six distinct color-coded clusters that collectively map the thematic architecture of AI research in pediatric radiology and medical imaging. The network reveals a field structured around two dominant methodological hubs deep learning and convolutional neural networks, which form the largest nodes in the red cluster and carry the highest total link strength across the entire network, confirming their role as the primary computational frameworks driving research output in this domain. Their co-occurrence with transfer learning, pneumonia, computer-aided diagnosis, chest x-ray, and feature extraction within the same cluster delineates a well-established research pipeline centered on CNN-based classification and detection tasks applied predominantly to chest imaging, reflecting the influence of landmark studies such as [36,42], which demonstrated the transferability of pre-trained deep learning models to pediatric diagnostic tasks. The green cluster groups artificial intelligence with radiography, radiology, bone age, scoliosis, and pediatrics, representing the clinical application layer of the field. The blue cluster anchored by machine learning occupies a bridging position between the deep learning hub and the clinical application cluster. The yellow cluster comprising MRI and ultrasound represents the two most clinically important non-ionizing imaging modalities in pediatric practice. The cyan and purple clusters together delineate the pediatric neuroimaging subfield, anchored by neuroimaging, neural networks, diagnosis, and autism spectrum disorder, which emerges as a relatively self-contained research community, with limited cross-cluster links to the broader imaging AI network.

### 3.10. Thematic Mapping of Pediatric Radiology and Medical Imaging Research

**Figure 6:**
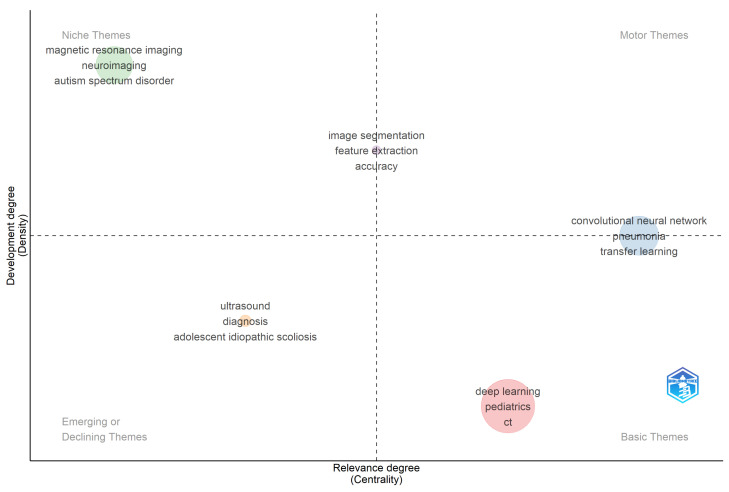
Thematic Mapping of Pediatric Radiology and Medical Imaging Research.

Figure 6 reveals the intellectual structure of AI research in pediatric radiology across four quadrants defined by the relative centrality and density of keyword clusters. Motor Themes (high centrality, high density), the intellectually dominant and well-developed core of the field, are represented in the revised analysis by the cluster comprising convolutional neural networks, pneumonia, and transfer learning, reflecting the maturation of CNN-based diagnostic pipelines, particularly in chest imaging applications. The positioning of this cluster as a Motor Theme indicates that CNN architectures have achieved both broad connectivity across the literature and strong internal methodological coherence. Niche Themes (low centrality, high density) include two distinct clusters: first, magnetic resonance imaging, neuroimaging, and autism spectrum disorder, representing a highly developed but relatively insular neuroimaging research community focused on pediatric brain development, and second, image segmentation, feature extraction, and accuracy, reflecting technically mature but narrowly scoped computer vision methodologies. These clusters are well developed internally but have not yet achieved the cross-field connectivity of Motor Themes, suggesting they remain somewhat siloed from the broader AI pediatric imaging literature. Basic Themes (high centrality, low density) include the cluster of deep learning, pediatrics, and computed tomography, which warrants specific interpretive comment. As noted above, ‘deep learning’ occupies this quadrant not because it is underdeveloped, it is the dominant methodology of the entire corpus, but because its near-universal co-occurrence with all other clusters produces a broadly distributed rather than internally cohesive connectivity profile. In thematic mapping methodology, terms that function as universal frameworks rather than distinct research directions characteristically produce this Basic Theme signature. This finding, therefore, reflects the foundational, cross-cutting role of deep learning in the field rather than any lack of methodological maturity. Emerging or Declining Themes (low centrality, low density) include ultrasound, diagnosis, and adolescent idiopathic scoliosis, indicating either newly emerging research directions or previously active topics losing momentum in the current literature.

## 4. Discussions

The bibliometric findings of this study collectively reveal a field that has grown rapidly, concentrated unevenly, and is now navigating the transition from technical proof-of-concept to responsible clinical integration. The following discussion interprets the key findings across five thematic dimensions.

### 4.1. Publication Growth and the Maturation of the Research Ecosystem

The exponential growth trajectory from 7 publications in 2005 to 559 in 2025 mirrors the development arc documented in other medical AI bibliometric analyses [20,30] but exhibits a notably steeper post-2018 inflection. Annual output remained below 20 publications until 2016, then accelerated sharply to 151 in 2020, 283 in 2022, 373 in 2023, and 559 in 2025, representing a nearly 80-fold increase over the two-decade span of the study. This acceleration is attributable to three structural drivers. First, the public release of large-scale pediatric imaging benchmarks exemplified by the RSNA Pediatric Bone Age Challenge dataset [46] substantially lowered the data-access barrier that had historically constrained pediatric AI research relative to adult imaging. Second, the maturation of transferable foundational architectures enabled researchers to adapt proven deep learning frameworks to pediatric-specific problems without building models from scratch, accelerating the pace of application-level research across multiple modalities and clinical conditions. Third, increasing interdisciplinary collaboration between computer scientists, radiologists, and pediatric clinicians generated a self-reinforcing cycle of methodological development and clinical validation, reflected in the growing presence of both engineering-oriented outlets such as IEEE Access (47 documents) and specialist clinical journals such as Pediatric Radiology (61 documents) in the source rankings, as shown in Table 2.

The dominance of journal articles (85.9%) over conference proceedings (14.1%) is interpretively significant and distinguishes pediatric imaging AI from the broader computer science literature, where conference proceedings traditionally carry greater methodological prestige. This publication pattern reflects the heightened ethical scrutiny applicable to AI systems deployed in vulnerable pediatric populations and the regulatory emphasis on peer-reviewed clinical validation prior to dissemination [10]. As Ref. [51] noted in their foundational survey of deep learning in medical image analysis, the transition from conference-driven to journal-driven dissemination is a hallmark of a field moving from exploratory prototyping toward clinically accountable research, a transition that this corpus clearly reflects.

### 4.2. Citation Architecture and Foundational Dependencies

The extreme citation skew observed in this corpus, with [36] accumulating 2886 citations, nearly five times the next-ranked paper, reflects a well-documented pattern in emerging applied AI fields, where early landmark feasibility demonstrations become obligatory reference anchors for subsequent work. Kermany et al.’s transfer learning framework, though not pediatric-specific in its original application, was rapidly adopted by pediatric imaging researchers precisely because it provided a validated, reproducible pipeline that could be fine-tuned on smaller pediatric datasets. This citation dominance signals both the field’s methodological pragmatism and its structural dependence on adult-data-derived models, a dependence that the thematic map analysis confirms has not yet been fully resolved, evidenced by the persistence of deep learning in the Basic Themes quadrant despite its methodological centrality.

The next tier of highly cited works reveals an important distinction. Zhang (616 citations) [37] on multimodal infant brain MRI segmentation and Smyser (572) [38] on preterm infant neural network development are genuinely pediatric-specific foundational contributions whose sustained citation influence over more than a decade indicates that early investments in pediatric neuroimaging infrastructure have generated compounding returns for the field. Their high citation counts stand in contrast to the majority of the top-cited list, which consists predominantly of general medical imaging papers subsequently adapted for pediatric use, a structural pattern that reveals how much of the field’s intellectual foundation is borrowed rather than built specifically for children.

The prominence of bone age assessment papers in the citation top tier by Lee (313 citations) [45], Halabi (304 citations) [46], and Spampinato (298 citations) [47] reflects the historical role of automated skeletal maturity estimation as the first widely validated clinical AI application in pediatric radiology. These papers established the field’s clinical credibility and continue to serve as methodological benchmarks. Notably, the inclusion of Kaissis (278 citations) [48] on privacy-preserving federated deep learning in the top-cited tier signals a growing awareness within the corpus of the data governance challenges unique to pediatric populations, where lifelong medical records and consent complexities demand technical solutions beyond conventional centralized training frameworks [52].

### 4.3. Source Landscape and the Interdisciplinary Character of the Field

The dual prominence of open-access megajournals Scientific Reports (87 documents) and PLOS ONE (50 documents) alongside specialist clinical outlets Pediatric Radiology (61 documents) and NeuroImage (54 documents) reflects a field navigating the tension between broad dissemination and rigorous clinical credentialing. Open-access venues accelerate visibility and citation uptake but may impose fewer methodological constraints than specialty journals with pediatric-specific peer review processes. The presence of NeuroImage (54 documents) and Human Brain Mapping (30 documents) among the top ten sources confirms that pediatric neuroimaging constitutes a substantial and self-sustaining research strand within the broader corpus, consistent with the niche positioning of MRI, neuroimaging, and autism spectrum disorder in the thematic map. The co-prominence of engineering-oriented venues IEEE Access (47 documents) and Biomedical Signal Processing and Control (31 documents) confirms that pediatric imaging AI remains an inherently cross-disciplinary enterprise, with significant methodological contributions from electrical engineering, signal processing, and computer vision. However, this interdisciplinary footprint creates a translational gap: engineering-led publications frequently optimize for benchmark performance metrics such as AUROC and Dice coefficient that may not directly correspond to clinically meaningful outcome measures in pediatric patients, including diagnostic accuracy improvement, radiation dose reduction, and workflow efficiency. The field’s most urgent need is not more methodological diversity but rather stronger mechanisms for translating technical achievements into verified clinical benefits.

### 4.4. Geographic and Institutional Concentration: Structural Inequity and Its Consequences

The geographic concentration of this corpus represents perhaps the most consequential finding of this bibliometric analysis. China dominates in absolute citation volume at 8804 TC, yet its average citation rate of 13.10 per article is the lowest among the top five, indicating that volumetric dominance does not translate into proportional per-article impact, consistent with findings from comparable medical AI bibliometric analyses [20,30]. A significant portion of Chinese output appears to be concentrated in technical algorithm development studies with limited international uptake, reflecting a research ecosystem optimized for output volume rather than cross-boundary clinical translation. The United States presents a striking inverse relationship: ranking only fifth in total citations (1782 TC) yet achieving the highest average citation rate at 26.10 per article. This indicates that U.S.-affiliated publications are selectively high-impact, focusing on landmark methodological contributions, benchmark dataset releases, foundational architecture papers, and multicenter clinical validation studies that attract disproportionately wide international citations. Papers originating from U.S. institutions, such as [36,46] with 2886 and 304 citations, respectively, exemplify this pattern. The United Kingdom and Germany, with identical average citation rates of 22.30, reflect the strength of European academic-clinical collaborations, particularly in MRI-based neuroimaging and radiation dose optimization, where both countries maintain well-established multicenter consortia.

At the institutional level, the concentration of output within eight North American academic medical centers in the top ten, led by Harvard Medical School (186 articles), the University of Toronto (165), and Stanford University (164), reflects structural advantages that compound over time: large pediatric patient populations, substantial research funding, and established clinical-research integration. The presence of the Children’s Hospital of Philadelphia (122 articles) as the only dedicated pediatric hospital in the top ten is particularly revealing. Its lower article count relative to research-intensive universities reflects a publication strategy oriented toward prospective clinical validation and translational impact, rather than algorithm-development volume, precisely the kind of contribution the field most urgently needs, but which the current citation structure undervalues.

Critically, the near-total absence of African, Middle Eastern, and Latin American countries from the top-ten list creates a structural misalignment between where knowledge is produced and where it is most urgently needed. These regions collectively bear a disproportionate burden of pediatric disease, yet contribute negligibly to the AI literature designed to address it. This is not merely an equity concern; it is a technical validity concern. AI models trained on homogeneous, high-resource imaging data from North American or European pediatric populations encode implicit assumptions about scanner hardware, imaging protocols, and disease prevalence distributions that may not hold in lower-resource settings. The bibliometric record thus reveals a field that may be systematically optimizing for performance in the settings where AI-assisted pediatric diagnosis is least urgently needed.

### 4.5. Thematic Architecture: From Technical Maturity to Clinical Integration

The keyword co-occurrence and thematic mapping analyses together reveal a field with a technically sophisticated but clinically incomplete knowledge base. The Motor Theme cluster convolutional neural network, pneumonia, and transfer learning demonstrates that the application of deep learning to pediatric chest imaging has achieved genuine methodological maturity, with well-validated pipelines and reproducible performance benchmarks established by studies such as [36,42]. The co-occurrence of transfer learning within this Motor Theme cluster is consistent with the field’s established practice of fine-tuning pre-trained models on smaller pediatric datasets, a pragmatic response to the chronic data scarcity that distinguishes pediatric from adult imaging AI research.

The two Niche Theme clusters with MRI, neuroimaging, and autism spectrum disorder in the first and image segmentation, feature extraction, and accuracy in the second represent areas of deep technical development that have not yet achieved the cross-field connectivity required to influence clinical practice broadly. The neuroimaging cluster, in particular, remains relatively insular, consistent with the self-contained nature of the pediatric neuroimaging research community, as evidenced by the co-occurrence network. The high citation counts of [38,39] within this cluster confirm that neuroimaging has a strong foundational base, but the limited cross-cluster connectivity of this niche suggests that advances in fMRI-based connectivity analysis and autism spectrum disorder imaging have not been systematically integrated with the broader deep learning methodological toolkit. Transformer-based architectures and graph neural networks, well-suited to connectivity modeling tasks, represent an underexplored methodological bridge between these communities.

The Basic Theme positioning of deep learning alongside pediatrics and computed tomography reflects its foundational, cross-cutting role as a universal computational framework rather than a distinct thematic research direction. As Ref. [34] notes, terms that function as universal frameworks rather than distinct research directions characteristically produce this Basic Theme signature because their near-universal co-occurrence distributes link weights too broadly to generate high density scores. This finding, therefore, reflects the dominance of deep learning as the field’s primary computational substrate rather than indicating methodological underdevelopment, an interpretation supported by its status as the largest node in the co-occurrence network (Figure 5) and its presence in the top-cited documents across multiple clinical application domains.

The Emerging or Declining Theme clustering ultrasound, diagnosis, and adolescent idiopathic scoliosis warrants careful interpretation. The declining centrality of ultrasound is unexpected, given its clinical importance as a radiation-free modality in pediatric practice, and may reflect that ultrasound AI research is increasingly conducted under more application-specific keyword terms, fragmenting its representation in the co-occurrence network rather than consolidating under the umbrella term. The presence of adolescent idiopathic scoliosis in this quadrant suggests that what was once a pioneering application domain for AI in pediatric imaging has been partially superseded by more technically sophisticated and broadly applicable approaches. Finally, the near-absence of governance, ethics, and explainability from the thematic map’s central quadrants signals that these considerations, despite their practical urgency for clinical deployment, remain structurally peripheral in the current literature [53]. Embedding ethical analysis and XAI requirements into AI development workflows from the outset represents one of the most pressing unmet needs in the field.

## 5. Limitations

This study has several inherent methodological limitations. First, the analysis is constrained by its data source, which relies exclusively on the Web of Science Core Collection, potentially missing relevant literature indexed in other databases. Second, the search strategy, though systematic, is based on a specific Boolean query and excludes non-English publications and document types beyond articles and conference papers, potentially introducing selection bias. Finally, the observed concentration of publications in high-output countries and institutions may be influenced by database indexing preferences rather than reflecting the full global distribution of research activity in pediatric imaging AI. Furthermore, author-level analyses are subject to name disambiguation limitations inherent to the WoSCC surname-initial indexing convention, which may lead to the aggregation of distinct researchers under identical name signatures and may disproportionately affect authors with common surnames.

## 6. Future Work

### 6.1. Construction of Geographically Representative Pediatric Imaging Datasets

The country-level analysis reveals that ten countries account for the overwhelming majority of total citations in the corpus, with China, the United States, the United Kingdom, Germany, and Canada alone responsible for the dominant share of both output and impact. Africa, Latin America, and most of Southeast Asia are structurally absent from the knowledge production map, contributing negligibly to the 2688 documents analyzed despite collectively bearing a disproportionate global burden of pediatric disease. The collaboration network reinforces this finding, showing that international co-authorship is concentrated along US-China and Europe-North America corridors, with low-income and middle-income country institutions largely outside these networks. These findings directly motivate the need for multicenter pediatric imaging cohorts specifically established in underrepresented regions, targeting conditions with high regional disease burden, including tuberculosis, malnutrition-related developmental disorders, and malaria-associated neurological sequelae that are largely absent from the current benchmark datasets. Federated learning frameworks, which appear in the corpus as an emerging research strand exemplified by the high-citation work, offer a technically viable route to pooling data across resource-constrained, privacy-sensitive settings without requiring centralized data transfer. Synthetic data generation using generative adversarial networks is a complementary strategy for augmenting rare-disease and rare-population datasets, particularly in pediatric oncology and neonatal imaging, where annotated data remain critically scarce.

### 6.2. Shift from Benchmark Performance to Prospective Clinical Validation

The most-cited contributions in pediatric radiology AI are predominantly technical feasibility studies rather than clinical outcome trials, exemplified by [36] and three bone age assessment papers that collectively account for thousands of citations. This pattern aligns with the thematic map, where technically mature computer vision methods (e.g., image segmentation, feature extraction) remain niche and have yet to integrate into clinical workflows. Consequently, the field requires a reoriented validation paradigm: future studies should prioritize pediatric-specific benchmarks, independent external validation, and reporting of clinical outcomes (e.g., radiologist efficiency, time-to-diagnosis, dose reduction) alongside conventional technical metrics. Integrating explainable AI and evaluating performance across diverse pediatric subpopulations are essential to build clinician trust and preempt disparities before deployment.

### 6.3. Diversification and Formalization of International Collaboration Networks

Co-authorship networks are concentrated along US–China, US–Europe, and intra-European corridors, with African, Latin American, and Southeast Asian institutions largely absent. Eight of the ten most productive affiliations are North American, and the only dedicated pediatric hospital in the top ten is the Children’s Hospital of Philadelphia, meaning the research agenda is shaped by institutions whose structural advantages do not reflect the pediatric healthcare challenges facing most of the world’s children. International professional societies should therefore establish targeted collaborations and funded programs to integrate underrepresented institutions into existing consortia, diversifying training data and building local AI capacity where clinical need is greatest. Future research should prioritize validating AI models across geographically diverse populations and developing sustained research infrastructure in underrepresented regions.

### 6.4. Methodological Integration of the Isolated Neuroimaging Cluster

The pediatric neuroimaging subfield—centered on neuroimaging, neural networks, diagnosis, and autism spectrum disorder, appears as a self-contained cluster in both the co-occurrence network and thematic map, with limited connections to the broader AI literature. Despite a strong foundational base, methodological advances such as transformers, graph neural networks, and self-supervised learning have yet to be systematically applied to pediatric functional neuroimaging. Bridging this niche with the CNN-based Motor Theme cluster by evaluating transformer and graph-based models for conditions like autism spectrum disorder, epilepsy, and cerebral palsy represents a key opportunity for expanding the field’s thematic core. Future research should therefore focus on systematically adapting these advanced architectures to pediatric neuroimaging applications to integrate the niche into the broader AI research landscape.

## 7. Conclusions

This study presents the first dedicated bibliometric analysis of AI applications in paediatric radiology and medical imaging, mapping the intellectual structure of a field that has produced 2688 publications over two decades and has grown from seven annual publications in 2005 to 559 in 2025. The analysis reveals not only what the field has produced but also the structural conditions that have shaped it and the structural gaps that now constrain its most important next steps.

Three findings stand out as analytically significant beyond their descriptive values. First, the field’s citation architecture is built on borrowed scaffolding. The most cited paper, led by [36] with 2886 citations, was designed for adult or general medical imaging and subsequently adapted for paediatric use. The paediatric imaging AI community has been methodologically fast-moving but intellectually dependent, rapidly deploying techniques developed elsewhere rather than building a body of foundational work specifically calibrated to the unique biological, ethical, and clinical demands of children. This is not a criticism of the field’s productivity; rather, it is a structural observation with direct implications for where original investment is most needed.

Second, the geographic and institutional concentration of knowledge production represents a structural risk that is simultaneously an equity and a scientific validity problem. When the overwhelming majority of a corpus is produced by institutions in five high-income countries, the implicit assumptions embedded in the resulting models about scanner quality, population demographics, disease prevalence, and imaging protocols reflect the environments in which those models were built, not the environments where they will have the greatest impact. The bibliometric record of this field does not merely show who is publishing; it reveals a systematic mismatch between the geography of knowledge production and the geography of paediatric health needs that no amount of methodological refinement can resolve without deliberate structural intervention.

Third, the thematic map reveals a field in which technical achievements and clinical ambitions are not yet aligned. The Motor Theme cluster CNN, pneumonia, and transfer learning represent genuine methodological maturity in a specific application niche: chest imaging for common paediatric conditions. However, this maturity is narrow. Neuroimaging and segmentation clusters, which address some of the most clinically complex and impactful applications of AI in paediatric medicine, remain peripheral, well-developed internally but insufficiently connected to the broader methodological mainstream to influence clinical practice at scale. Governance, ethics, and explainability, the preconditions for the responsible deployment of any of these methods in children, are absent from the thematic centre entirely.

Taken together, these three findings describe a field that has successfully demonstrated what AI can do in paediatric radiology but has not yet built the conditions necessary for AI to do what paediatric radiology needs. Closing that gap will require not faster algorithms or larger benchmarks but rather a deliberate reorientation of the field’s investment priorities toward geographic inclusion in data collection, clinical outcome validation rather than benchmark optimisation and the governance infrastructure that responsible deployment in vulnerable populations demands. This bibliometric analysis provides an empirical basis for reorientation by mapping not only where the field stands but precisely where its structural logic must change.

## Figures and Tables

**Table 1:** Search string used for topic retrieval (Web of Science).

Field	Search String
AI Methods	“artificial intelligence” OR “machine learning” OR “deep learning” OR “neural network” OR “convolutional neural network” OR CNN OR “transformer model” OR “computer-aided diagnosis” OR “automated detection” OR “supervised learning” OR “unsupervised learning” OR “self-supervised learning”
Pediatric Population	pediatric OR paediatric OR child OR infant OR newborn OR neonate OR adolescent OR “young patient” OR “pediatric population” OR “paediatric population”
Medical Imaging	“medical imaging” OR radiology OR “diagnostic imaging” OR “image analysis” OR “radiographic imaging” OR “CT scan” OR “computed tomography” OR MRI OR “magnetic resonance imaging” OR ultrasound OR sonography OR “nuclear imaging” OR “X-ray” OR radiograph OR “image segmentation” OR “lesion detection” OR “image classification” OR radiomics

**Table 2:** Most Relevant Sources.

Source	No. of Documents
Scientific Reports	87
Pediatric Radiology	61
NeuroImage	54
PLOS ONE	50
IEEE Access	47
Diagnostics	39
Biomedical Signal Processing and Control	31
Frontiers in Pediatrics	31
Human Brain Mapping	30
Frontiers in Neuroscience	29

**Table 3:** Most Relevant Authors.

Author	No. of Documents
Zhang Y	68
Wang Y	60
Wang L	50
Li J	47
Li H	45
Liu Y	45
Wang J	43
Zhang J	42
Li Y	38
Chen Y	37

**Table 4:** Top Cited Documents.

Paper	DOI	Total Citations
Kermany DS, 2018 [36]	10.1016/j.cell.2018.02.010	2886
Zhang W, 2015 [37]	10.1016/j.neuroimage.2014.12.061	616
Smyser CD, 2010 [38]	10.1093/cercor/bhq035	572
Batterink L, 2010 [39]	10.1016/j.neuroimage.2010.05.059	435
Mardani M, 2019 [40]	10.1109/TMI.2018.2858752	417
Dolz J, 2019 [41]	10.1109/TMI.2018.2878669	404
Chouhan V, 2020 [42]	10.3390/app10020559	374
Larson DB, 2018 [43]	10.1148/radiol.2017170236	330
Carnell S, 2012 [44]	10.1111/j.1467-789X.2011.00927.x	330
Lee H, 2017 [45]	10.1007/s10278-017-9955-8	313
Halabi SS, 2019 [46]	10.1148/radiol.2018180736	304
Spampinato C, 2017 [47]	10.1016/j.media.2016.10.010	298
Kaissis G, 2021 [48]	10.1038/s42256-021-00337-8	278
Jaiswal AK, 2019 [49]	10.1016/j.measurement.2019.05.076	261
Liang G, 2020 [50]	10.1016/j.cmpb.2019.06.023	258

**Table 5:** Most Productive Countries’ Distribution.

S/N	Country	TC	Average Article Citations
1	China	8804	13.10
2	United Kingdom	2363	22.30
3	Germany	2213	22.30
4	Canada	2102	15.00
5	USA	1782	26.10
6	Korea	1308	11.30
7	India	1007	9.30
8	Australia	865	21.00
9	France	834	18.10
10	Spain	713	18.00

**Table 6:** Most Relevant Affiliation Distribution.

S/N	Affiliation	Number of Articles
1	Harvard Medical School	186
2	University of Toronto	165
3	Stanford University	164
4	University of Pennsylvania	156
5	Children’s Hospital of Philadelphia	122
6	University of Calgary	115
7	Shanghai Jiao Tong University	106
8	Zhejiang University	106
9	Washington University	103
10	University of North Carolina	99

## Data Availability

The data presented in this study are available upon request from the corresponding author.

## Abbreviations

The following abbreviations are used in this manuscript:

AI	Artificial Intelligence
ASD	Autism Spectrum Disorder
CNN	Convolutional Neural Network
CT	Computed Tomography
DL	Deep Learning
fMRI	Functional Magnetic Resonance Imaging
GPU	Graphics Processing Unit
MRI	Magnetic Resonance Imaging
PLOS	Public Library of Science
RSNA	Radiological Society of North America
SCIE	Science Citation Index Expanded
TC	Total Citations
TS	Topic Search
WoSCC	Web of Science Core Collection
XAI	eXplainable Artificial Intelligence
